# On-the-fly selection of cell-specific enhancers, genes, miRNAs and proteins across the human body using SlideBase

**DOI:** 10.1093/database/baw144

**Published:** 2016-12-26

**Authors:** Hans Ienasescu, Kang Li, Robin Andersson, Morana Vitezic, Sarah Rennie, Yun Chen, Kristoffer Vitting-Seerup, Emil Lagoni, Mette Boyd, Jette Bornholdt, Michiel J. L. de Hoon, Hideya Kawaji, Timo Lassmann, The FANTOM Consortium, Yoshihide Hayashizaki, Alistair R. R. Forrest, Piero Carninci, Albin Sandelin

**Affiliations:** 1 Department of Biology, The Bioinformatics Centre, University of Copenhagen, Ole Maaloes Vej 5, Copenhagen N, DK2200, Denmark; 2 Biotech Research and Innovation Centre, University of Copenhagen, Ole Maaloes Vej 5, Copenhagen N, DK2200, Denmark; 3 Department of Mathematical Sciences, University of Copenhagen, Universitetsparken 5, Copenhagen Ø, DK2100, Denmark; 4 RIKEN Center for Life Science Technologies (Division of Genomic Technologies), 1-7-22 Suehiro-cho, Tsurumi-ku, Yokohama 230-0045, Japan; 5 RIKEN Preventive Medicine and Diagnosis Innovation Program, 1-7-22 Suehiro-cho, Tsurumi-ku, Yokohama 230-0045, Japan; 6 Telethon Kids Institute, The University of Western Australia, 100 Roberts Road, Subiaco, 6008, Australia Western Australia; 7 Harry Perkins Institute of Medical Research, QEII Medical Centre and Centre for Medical Research, the University of Western Australia, Nedlands, Western Australia, Australia

## Abstract

Genomics consortia have produced large datasets profiling the expression of genes, micro-RNAs, enhancers and more across human tissues or cells. There is a need for intuitive tools to select subsets of such data that is the most relevant for specific studies. To this end, we present SlideBase, a web tool which offers a new way of selecting genes, promoters, enhancers and microRNAs that are preferentially expressed/used in a specified set of cells/tissues, based on the use of interactive sliders. With the help of sliders, SlideBase enables users to define custom expression thresholds for individual cell types/tissues, producing sets of genes, enhancers etc. which satisfy these constraints. Changes in slider settings result in simultaneous changes in the selected sets, updated in real time. SlideBase is linked to major databases from genomics consortia, including FANTOM, GTEx, The Human Protein Atlas and BioGPS.

**Database URL:** http://slidebase.binf.ku.dk

## Background

Large consortia such as ENCODE (1), The Human Protein Atlas (2), Functional Annotation of Mammals (FANTOM) (3) and Genotype-Tissue Expression (GTEx) (4) have measured the molecular states of cells and tissues across the human body on different functional levels, including the expression of genes, miRNAs, enhancer regions and the abundance of proteins.

While these atlases represent powerful resources for understanding of human biology and disease, the navigation of such vast datasets is daunting for the majority of potential users. While several resources are available for showing the expression of a selected gene across multiple experiments of states [e.g. Bloodspot (5), Expression Atlas (6), BioGPS(7)], there is a lack of intuitive methods for selecting sets of genes (or, depending on data, proteins, miRNAs, enhancers etc.) that are specifically used within tissues or cells of interest. This presents a bottleneck for the broader usage of the data by the wider community.

Although pre-defining feature sets (e.g. ‘liver-specific genes’) is possible, usage requirements typically vary from case to case, and there are many ways of defining such sets. Complicating the selection process, tissue-exclusive expression for a given gene is rare, and related to this, many users require joint expression in two or more tissues/cells (e.g. ‘expression must come from mostly blood and brain’) or the selection of more complex sets where expression of some tissues are allowed but others not (e.g. ‘expression in epithelia cells but not immune cells’). A related issue which makes computational/statistical methods for selection challenging is that samples or conditions in most datasets are not equidistant from each other [e.g. brain tissues are often distinct but yet more similar to each other than other organs (8)].

Therefore, thresholds for cell- or tissue specificity are best defined by the users themselves in an iterative and exploratory fashion. User-friendly tools for this process are currently lacking.

To this end, we present SlideBase (http://slidebase.binf.ku.dk), a web-based tool interfacing leading genomics resources, which enables users to select genes, enhancers, promoters, miRNAs with user-customisable expression thresholds for each sample, through the usage of interactive sliders.

## Results

### Selecting genomic features by expression constraints defined by sliders

In SlideBase we aimed to create a simple and intuitive interface for users to search for genomic features (e.g. genes, enhancers) based on their expression across different samples (e.g. heart, brain, liver) from a given experimental resource [such as FANTOM (3), GTEx (4) or The Human Protein Atlas (2)].

**Figure 1.**
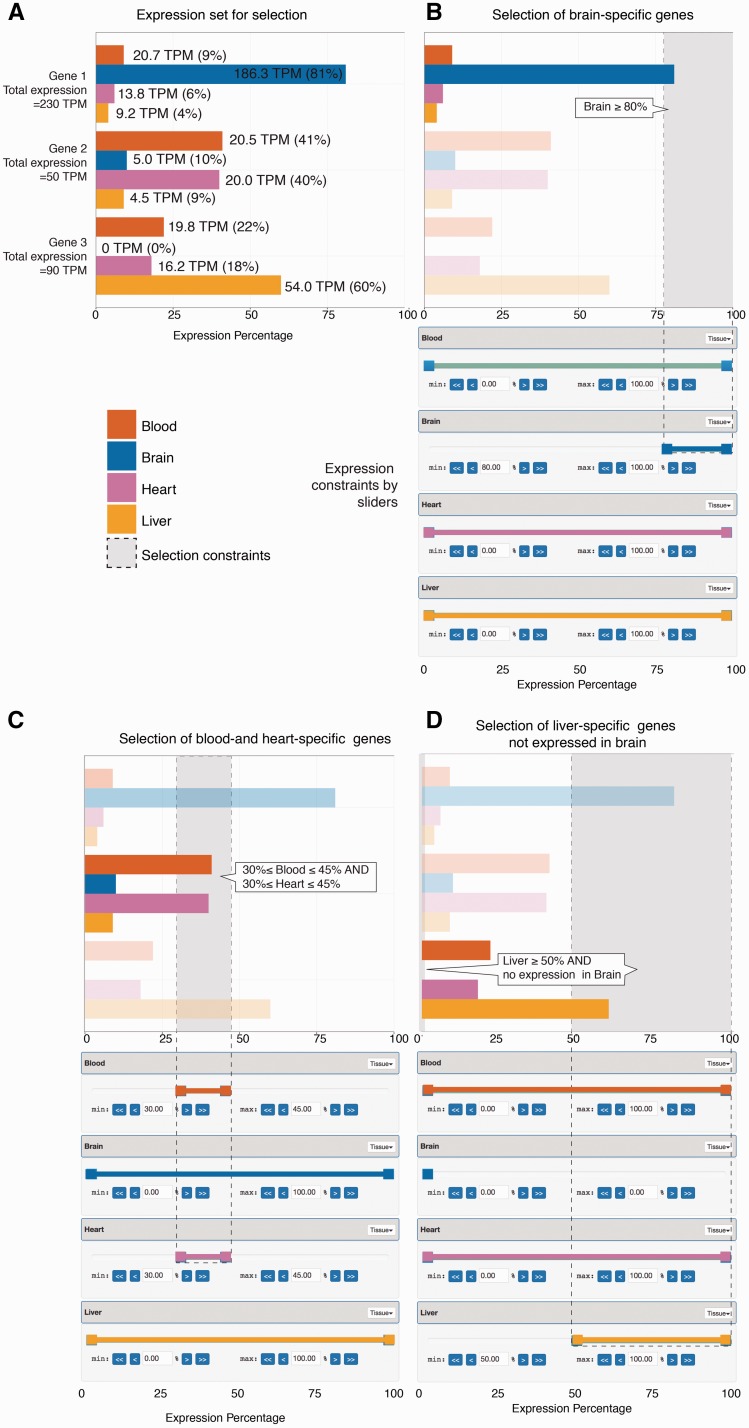
Selecting genes based on expression levels using sliders. The figure shows a simple artificial example based on three genes and expression data across four tissues (shown in panel A), and three example selections based on sliders shown beneath each bar plot (panels B–D), where the bar plots show the results of each slider configuration. (A) *Expression set for selection*: The bar plot shows the expression levels of three example genes across four samples (blood, brain, heart and liver) from CAGE data, as indicated by colour. While basal expression values originate from CAGE data, and are measured as TPM (tags per million, values shown at each bar), these are normalized so that the contribution from each tissue sums to 100% per gene (shown on *X* axis). (B) *Selection of brain-specific genes using sliders*: For each of the four tissues, we create a percentage ‘slider’, shown beneath the bar plot, with colours corresponding to respective tissue. Each slider is interactive and is used to define a percentage interval of expression thresholds. Having a slider for each sample allows the user to specify the amount of expression originating from one or more samples. In doing so, the results will only contain genes which comply with the described constraints. In this example, to select gene(s) that are predominantly expressed in brain, the left handle of the brain slider is moved to 80% while the right handle remains at 100%. As a result we define the percentage interval [80%, 100%] for brain, which is equivalent to having at least 80% and at most 100% of gene expression come from brain. The selected constraints are shown as dotted lines and a grey area within the bar plot, and identified by a callout. Only gene 1 satisfies these constraints (genes not satisfying the constraints are translucent). (C) *Selection of blood- and heart-specific genes*: Bar plots and sliders are organized as in panel B. In addition to setting constraints for a single slider it is also possible to combine multiple sliders to create a more refined search. In this example, we require that at least 30% and at most 45% of the expression comes from blood. Furthermore, we also require that another minimum of 30% and maximum of 45% of expression comes from heart. We achieve this by moving the blood and heart left slider handles to 30% and right slider handles to 45%. Only gene 2 satisfies these constraints. (D) *Selection of liver-specific genes not expressed in brain*: Bar plots and sliders are organized as in panel B. In this example, we wish to select genes, which have no expression in brain, but have at least 50% expression from liver. In order to require that no expression originate from brain, both slider handles are moved to 0% (at least 0% and at most 0%). Finally, we move the liver left slider handle to 50%. Only gene 3 satisfies these constraints.

Given the expression of genomic features (such as genes) across samples, we transformed expression values into percentage of expression values in each individual sample, relative to all samples. As a simple example, suppose we have expression values from CAGE data, defined as Tags Per Million (TPM) for a set of three genes across four samples: brain, blood, heart and liver. These values are normalized per gene basis to sum to 100% and the normalized values will be regarded as the contribution of expression from each sample per gene basis (Figure 1A).

In order to select genes based on these values, we define a ‘slider’ with values ranging from a minimum of 0% to a maximum of 100% for each sample. Each such slider works as an interactive expression contribution threshold setting device, effectively defining how much of expression that is required to come from e.g. brain. This is done by moving two percentage handles, which is equivalent to setting expression contribution thresholds for minimal or maximal expression.

For example, moving the ‘min’ threshold slider to 80% in the brain slider will select only genes for which brain contributes at least 80%—which in this case is only true for gene 1 (Figure 1B). In addition, slider constraints across many samples can be combined, so that we can request a specific contribution from e.g. blood and heart (Figure 1C). Moreover, sliders offer the option to exclude genes expressed in specified samples by setting both slider handles to 0%; this can be combined with other constraints to further refine the search (Figure 1D).

**Figure 2.**
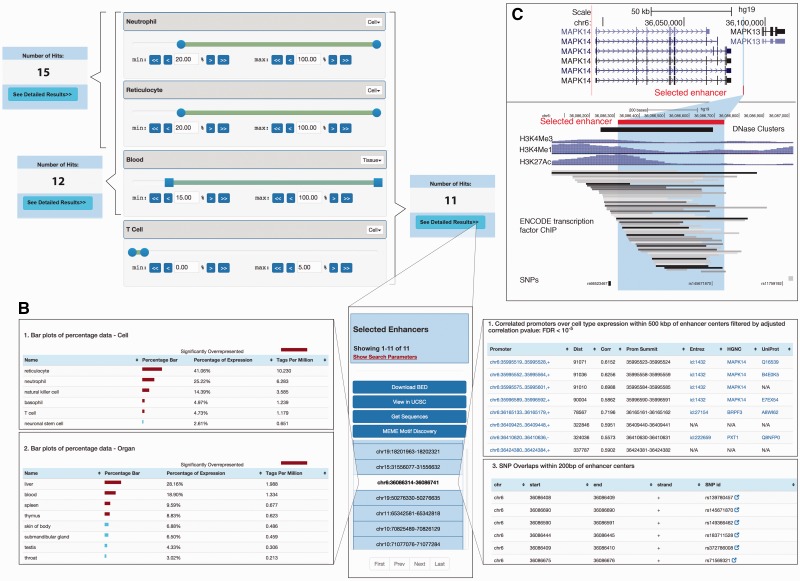
Example of selection of tissue-specific enhancers. The selection of genes, proteins or miRNAs based on expression or abundance works similarly. (A) *Selection of cell/tissue-specific enhancers based on sliders*: Sliders for neutrophils, reticulocytes and T cells as well as whole blood are shown (out of a total of 69 cell + 41 tissue sliders, where slides that are not shown are set to defaults = no constraints). The number of selected enhancers obtained from moving sliders is shown in blue boxes. Selecting a minimum expression contribution of 20% from neutrophils and reticulocytes results in 15 enhancers. If also requiring ≥15% expression from blood, the number of enhancers decreases to 12. Sliders also allow for negative selection, allowing at maximum only a certain amount of expression from a cell type: this is done by the right slider handle, exemplified by permitting at maximum 5% T-cell expression. The 11 enhancers resulting from the overall selection are shown in panel B. (B) *Detailed expression of selected enhancers*. *Middle*: Overview of the enhancers selected in panel A. The highlighted enhancer serves as an example for data in left and right panels. *Left*: Detailed expression data across all FANTOM5 samples for each enhancer for tissues/organs and primary cells. *Right*: SNP overlap and predicted promoter–enhancer associations of selected enhancers. Note that not all data present in the web tool is shown. (C) *UCSC browser views of the enhancer region highlighted in panels B.* Upper panel shows the larger gene landscape, including the *MAPK14* gene linked to the enhancer highlighted in panel B. Lower panel shows a zoom-in.

As an applied example showing the abilities of SlideBase with real data, let us assume we wish to select enhancer regions used in neutrophils, reticulocytes and whole blood from the FANTOM5 body-wide human enhancer atlas (9) (Figure 2). The atlas measures enhancer RNA expression across cells and tissues, which is a proxy for enhancer activity (9). In order to select an enhancer where at least 20% of its expression originates from neutrophil cells, the left neutrophil slider handle is moved to ≥20%. To additionally require at least 20% expression from reticulocyte cells, the corresponding slider handle is moved to ≥20%; we also require that at least 15% expression originate from blood (Figure 2A). The number of selected enhancers satisfying these criteria is updated in real time as slider bars are moved (Figure 2A)—the selection above results in 12 enhancer regions, out of a total of ∼33 000 regions. It is also possible to add negative selections by moving the right-most slider handle. For instance, to additionally require that not more than  5% of the total expression derive from T-cells, we move the T-cell right slider handle to ≤5% (Figure 2A). This reduces the number of resulting enhancers to 11.

Once a selection has been made, the expression levels of selected enhancers are shown across all FANTOM samples (Figure 2B, left panel), along with overlaps with SNPs and likely target genes predicted by co-expression (9) (Figure 2B, right panel). Selected enhancers can be visualized in genome browsers with a simple click (Figure 2C). Selected enhancer regions may also be downloaded as BED and FASTA files, or analyzed for DNA motifs. For a detailed discussion on selection features, see below.

### SlideBase datasets

We have built SlideBase tools to navigate a range of different genomic entities measured across human tissues or cell types based on different technological platforms: micro-array-based gene expression data from BioGPS (7), miRNA expression based on small RNA sequencing (deHoon *et al*., under review), transcription start site and enhancer RNA expression by CAGE (3,9), RNA-seq-based gene expression from GTEx (4) and joint RNA-seq expression and protein abundance using antibody-based immunohistochemical staining from the Human Protein Atlas (2) (Table 1). While the large majority of the web features showcased in Figure 2 for enhancer regions are available for all datasets, each selection tool is customized to fit the underlying data, and to link back to the original resource once the features of interest have been selected. Also, depending on the source data, specific selection modes are available, outlined below. For instance, for the Human Protein Atlas data, it is possible to place constraints on both protein abundance and RNA expression levels simultaneously.

**Table 1.** Current datasets within SlideBase

Expression features	# Features for selection	# Cell or tissue types	Experimental technique	Underlying database/resource
Gene expression	24 602 genes	84 tissues and primary cells	Microarray	BioGPS (7)
Gene expression	19 692 genes	32 tissues	RNA-seq	Human protein atlas (2)
Gene expression	41 991 genes	53 tissues	RNA-seq (median expression across large human cohorts)	GTEx (4) (v6)
Transcription start site expression	184 476 TSSs	69 primary cell groups, 41 tissue groups	CAGE	FANTOM promoter atlas (3)
Enhancer RNA expression	32 693 enhancers	69 primary cell groups, 41 tissue groups	CAGE	Human enhancer atlas (9)
miRNA expression	1857 miRNAs	67 primary cells	sRNA-seq	Data described in de Rie *et al.*
Protein expression	14 578 proteins	45 tissues	Immunohisto-chemical staining	The human protein atlas (2) (v15)

### SlideBase selector tools

For each dataset, we have created and adapted a specific selector tool. The selector tools for the five datasets are very similar in their functionality—therefore, we have chosen to describe one tool (the enhancer selector) in detail and then highlight the differences for the other datasets and corresponding selector tools. The main components for a selector tool are the ‘search’ (or ‘selector’) page and the ‘viewer’ page. The search page contains sliders for the samples that underlie the specific dataset, as well as custom search options that fit respective data sets, such as searching for specific gene names or genomic regions, as applicable to respective set. The viewer page displays an overview of the returned results, which includes expression on detailed level, along with other information and options. Complementing the below description of respective sets, tutorial videos, and the more detailed documentation pages are available at the homepage.

*Human enhancer selector***.** The human enhancer selector (Figure 2A) enables searching for human enhancers (9) based on enhancer RNA expression in primary cells and/or tissues. Two sets of sliders are available corresponding to 69 primary cells groups and 41 tissue groups. Each such slider works as in the example in Figures 1 and 2. The two slider sets can be used independently or in a combined fashion, corresponding to either an ‘*OR*’ or ‘*AND*’ statement in selection. After adjusting the percentage values, the number of enhancers that satisfy the selected constraints are shown in the left upper corner or in a popup window. The number of resulting enhancers is updated in real time based on how constraints are changed. After a satisfying set of thresholds is selected, a detailed view of resulting enhancers can be accessed by pressing the ‘See Detailed Results’ button.

The primary cell and organ percentage constraint values can be disabled by clicking the ‘Disable’ button located in the header of both slider set containers. Disabling the percentage values for either or both cells and tissues implies that their percentage numbers will not be taken into account when searching for enhancers.

The ‘Viewer page’ (Figure 2B) displays detailed information about the resulting enhancers and allows the user to perform actions on the resulting set such as downloading and viewing in genome browsers. Clicking on an enhancer shows information about that individual enhancer as three different panels (tabs) ‘CAGE Details’ for expression, ‘Browse’ for genome browser views and ‘View Overlaps’ for basal overlaps and correlations with proximal genomic entities.

The CAGE detail panel shows the enhancer expression. Specifically, percentage and expression tables for primary cells/organ groups [called facets in the original article (9)], and libraries (actual sample level expression for all the samples that make up each facet) are shown (Figure 2B, left panel). Red bars indicate that a particular sample is statistically over-represented as defined in Ref. (9). The expression table contains links to the FANTOM SSTAR resource (3,10) which holds detailed information about each sample.

In the browser panel, the user can create a custom track of selected enhancers in the UCSC genome browser (Figure 2C) (11) or look them up in the ZENBU genome browser (12).

The ‘View overlaps’ panel contains predicted target promoter(s) of selected enhancers [predicted by expression correlations as in Ref. (9)], and SNP overlaps from dbSNP (13) (Figure 2B, right panel). If an enhancer–promoter overlap exists, the TSS regions can be further inspected by clicking on the promoter annotation, which will take the user to the corresponding SlideBase promoter viewer page. An enhancer-SNP overlap is reported when the distance between the enhancer midpoint and the SNP is 200 bp or less. Overlapping SNPs are linked to dbSNP (13).

In addition to selecting a single enhancer, a number of options are available for the whole set of resulting enhancers. The options are displayed as buttons shown above the enhancer list, and enable (i) downloading the coordinates of selected enhancers, (ii) uploading all selected enhancers as a track in UCSC Genome Browser (11) (similar to above, but the whole set of selected enhancers will create a single UCSC track), (iii) obtaining the DNA of selected enhancers (with optional flanks) as a FASTA file and (iv) motif discovery of these DNA sequences using MEME (14).

**Figure 3.**
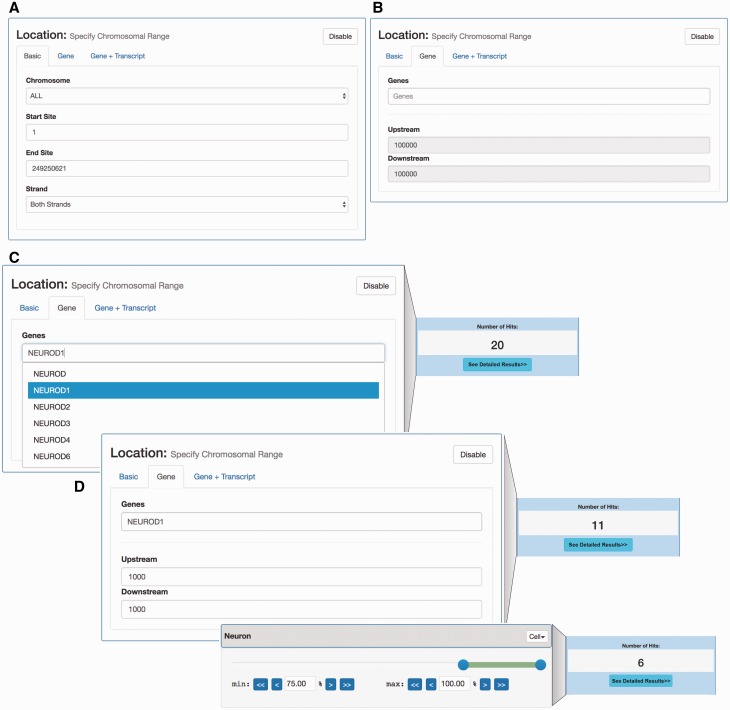
Location- and expression-based promoter search. (A) *Interface for selecting CAGE-defined promoters localized in a given genomic region*. (B) *Interface for selecting promoters localized around a given gene*. This interface allows for the selection of the set of CAGE-defined promoters that localize within a certain window around an annotated gene TSS. (C) *Example of gene-based selection of promoters*. *Left panel*: Using the gene-based search interface in panel B, the *NEUROD1* gene was selected: the dropdown menu suggests official gene names matching user input. As a default, all CAGE promoters 100 kbp around the UCSC-gene annotated TSS for *NEUROD1* are selected, which results in 20 promoters (right panel) (D) *Example of combined gene and tissue/cell constraint-based selection*. The upper left panel exemplifies a more focused selection of CAGE promoters by constraining the genomic region analysed to ± 1000 bp of the annotated *NEUROD1* TSS. This results in the selection of 11 promoters (right upper panel). On top of this, we add an additional expression constraint using a slider, where at least 75% of expression must come from neuron samples (lower left panel). This results in a subset of 6 promoters (lower right panel), compared to the 11 selected above.

*Human promoter selector*. The human promoter selector is based on clusters of CAGE tags, sampled across a wide range of tissues and primary cells in the FANTOM5 project (3,15). As CAGE tags primarily identify 5′ ends of RNAs, clusters of CAGE tags identify transcription start sites and thereby core promoter locations and expression. The data set covers the same cells and organs as in the enhancer data set described above. Because of this, the promoter search page has the same sample sliders and offers the same search and filter functionality as the enhancer search page. In addition to expression-based filtering, it is also possible to use a genomic location-based filter. The location search offers the option to search for promoters at any specific genomic location (Figure 3A) or at genomic locations given by known genes (Figure 3B). Furthermore, the user is able to combine location search with expression constraints in order to create more precise filters (exemplified in Figure 3C).

Similar, the corresponding viewer page is almost identical to the enhancer viewer page, but shows additional annotation details for promoters. In particular, a ‘promoter annotation’ tab is available and specifies if the promoter is associated with known genes or is novel. If the promoter is associated with a known gene [as defined in Ref. (3)], links to NCBI Gene (16), UniProt (17) and FANTOM SSTAR (10) databases are available.

*Human miRNA selector*. The miRNA selector allows for searching for miRNAs expressed across 67 human cell types, based on small RNA-seq (de Rie *et al*., submitted). Therefore, only a single panel of sliders is available within this SlideBase selector.

The miRNA viewer page is almost identical to the enhancer viewer page offering options for viewing miRNAs expression, downloading as BED and FASTA files, and browsing miRNAs locations in the UCSC Genome Browser.

*GTEx selector*. The GTEx project is a large-scale effort to profile gene expression in a wide range of tissues and individuals using RNA-seq in combination with genotyping of the same individuals, enabling the detection of quantitative trait loci (4). In SlideBase, we were interested in overall expression patterns across tissues rather than genetics. Therefore, we created sliders corresponding to the median expression across individuals for 53 tissues in GTEx. The viewer page displays expression levels across different samples for the resulting genes, displays linked single tissue eQTLs, and allows the user to perform actions on the resulting set such as viewing genes in the GTEx Gene Page and GTEx eQTL Browser along with viewing linked eQTLs in the GTEx SNP Page.

*BioGPS GeneAtlas selector*. BioGPS is a gene information portal, which can be used to make custom reports on genes and expression (7). One of the most popular features of BioGPS is the expression atlas, based on systematic microarray analysis of cells and tissues using U133A Affymetrix arrays.

Based on the expression of individual genes across different samples, there are a total of 84 sliders for the dataset samples which include both tissues and primary cells, allowing the selection of Affymetrix probe sets (typically corresponding to annotated genes) with custom expression thresholds.

The viewer page displays information about the resulting probe sets, their expression across samples, linked genes and allows the user to perform actions on the resulting set such as viewing genes in the BioGPS Gene Report page on the BioGPS website.

**Figure 4.**
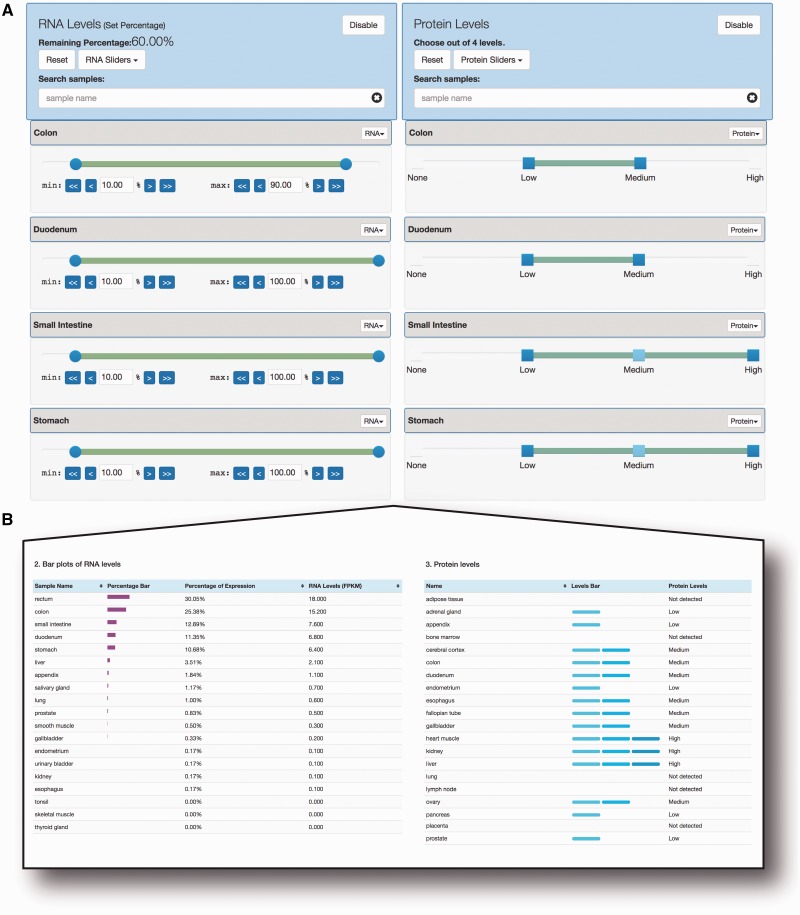
Dual selection of RNA and protein levels using sliders. (A) *Slider-based selection of RNA and protein levels*. Using data from the Human Protein Atlas, SlideBase uses pairs of sliders for RNA levels (left) and protein levels (right). Simultaneous constraints on matched tissues on RNA and protein level can be applied, and the resulting number of genes is updated in real time. The sliders work as in Figure 1, but based on the nature of the underlying data, protein levels are divided into four set categories. (B) *Example of output from the search constraints in panel A*. RNA expression and protein abundance are shown in left and right panel, respectively. Note that all output is not shown due to size constraints (in total, data from 32 and 45 tissues for RNA and protein levels, respectively, are available).

*Human Protein Atlas selector*. The human protein atlas combines transcriptomics data (RNA-seq) and immunohistochemistry data on protein level from a wide range of cell types and tissues, for the large majority of human protein coding genes (2). For this data, SlideBase uses pairs of sliders corresponding to transcript and protein abundance constraints across 32 tissue/cell types. In this way, it is possible to select for genes which are both expressed and translated in a given cell type, or any other combination (Figure 4A). While the RNA-seq sliders are conceptually identical to that of CAGE or microarray data above, the protein sliders are based on four step criteria [‘None’ (not detected), ‘Low’, ’Medium’ and ‘High’], as defined by the underlying resource.

The viewer page displays information about the resulting genes, their expression and protein abundance (Figure 4B), and allows the user to perform actions on the resulting set such as viewing genes in The Human Protein Atlas web tool.

## Implementation

SlideBase was developed as a web user interface using HTML for displaying pages, CSS/Bootstrap (http://getbootstrap.com) for styling and JavaScript along with JavaScript-based libraries to support interactive page components such as sliders. The server side component was implemented using the Laravel PHP framework (https://laravel.com/) for application logic along with a MySQL database (https://www.mysql.com) for data handling. The server also employed an Opal web service client (18) for MEME jobs.

## Discussion

While the current implementation of SlideBase demonstrates the clear utility of the slider selection concept for diverse data sets, SlideBase selectors will also be enabled for other data types in collaboration with the community. This could go beyond expression-based data. For instance, it is easy to imagine slider-based selection on data split by disease- or trait-specificity rather than cell or tissue, or selection based on other features than expression, such as the density of sequence patterns. In the future, we will also develop a system in which custom slider sets can be created to fit a new dataset submitted by users. In such a setting, SlideBase would be used as an exploratory tool in parallel with data production rather than being based on existing resources.

## Dataset links

FANTOM5 Promoters: http://fantom.gsc.riken.jp/data/ FANTOM5 Enhancers: http://fantom.gsc.riken.jp/data/ FANTOM5 miRNAs: http://fantom.gsc.riken.jp/data/ BioGPS Dataset: GeneAtlas U133A, gcrma: http://biogps.org/dataset/GSE1133/geneatlas-u133a-gcrma/

Human Protein Atlas: http://v15.proteinatlas.org/about/download, http://v15.proteinatlas.org/download/normal_tissue.csv.zip, http://v15.proteinatlas.org/download/rna_tissue.csv.zip, http://v15.proteinatlas.org/download/proteinatlas.xml.gz

GTEx V6: Register a free account at gtexportal.org to download these files. For RNA-seq, the GTEx Analysis V6 gene median dataset was used (GTEx_Analysis_v6_RNA-seq_RNA-SeQCv1.1.8_gene_median_rpkm.gct.gz). For eQTL SNP annotation, we used (GTEx_ Analysis _V6_ eQTLs.tar.gz).
